# Bacteria *Pseudomonas* sp. and *Pantoea* sp. Are the New Etiological Agents of Diseases on Forest Trees

**DOI:** 10.3390/plants14040563

**Published:** 2025-02-12

**Authors:** Elena Porotikova, Natalia Brusnova, Andrei Sushchenko, Galina Kolganikhina, Svetlana Vinogradova

**Affiliations:** 1 Institute of Bioengineering, Research Center of Biotechnology of the Russian Academy of Sciences, Leninsky Prospect 33, 119071 Moscow, Russia; plantvirus@mail.ru (E.P.); brusnova.natasha@yandex.ru (N.B.); andreysusch2000@mail.ru (A.S.); 2 Institute of Forest Science, Russian Academy of Sciences, 143030 Uspenskoe, Moscow Region, Russia; kolganihina@rambler.ru

**Keywords:** *Acer tataricum*, *Ulmus laevis*, *Ulmus minor*, *Fraxinus pennsylvanica*, bacterial pathogens, Tajima statistics, necrotic pathogens, dark wood

## Abstract

Forest trees significantly affect human life. The spread of pathogens, including bacterial ones, poses a serious threat to their health. Despite this, however, the species composition and distribution of pathogenic bacteria, as well as the etiology of common diseases affecting forest trees, remain virtually unstudied. In this study, we, for the first time, describe different species of *Pseudomonas* and *Pantoea* as new etiological agents associated with the symptoms of leaf spotting and wood darkening on *Acer tataricum* L., *Fraxinus pennsylvanica* L., *Ulmus minor* Mill. *Ulmus laevis* Pallas. and *Populus tremula* L. For the identification of bacteria species, we used an integrated approach based on the characterization of their morphology, biochemistry, physiology and genetics. Phylogenetic analysis was performed using multilocus typing for five genes for *Pseudomonas* and six genes for *Pantoea*. Leaf spotting on *A. tataricum*, *F. pennsylvanica*, *U. minor* and *U. laevis* was shown to be caused by *Pseudomonas cerasi*, *Pseudomonas congelans*, *Pseudomonas graminis*, *Pseudomonas syringae* and *Pantoea agglomerans* both in monoinfection and coinfection. Wood darkening in *U. minor U. laevis* and *P. tremula* was found to be associated with the presence of *Pantoea* sp. and *P. agglomerans*. The coinfection of forest trees with bacteria of the genera *Pseudomonas* and *Pantoea* indicates a complex mechanism of interaction between the two populations, which will be the subject of future studies.

## 1. Introduction

Forests are dynamically developing ecosystems that have a huge resource potential and play a vital role in maintaining the ecological, economic and social aspects of human life [1,2]. According to the global assessment of forest resources, the total area of forests in the world is slightly over 4 billion hectares and occupies about 31% of the total land area [3]. More than half of all forests are concentrated within the territory of five countries: the Russian Federation (815 million hectares), Brazil (497 million hectares), Canada (347 million hectares), the United States of America (310 million hectares) and China (220 million hectares) [3].

The economic significance of forest plantations lies in the role of forest resources in the production of building materials, paper, biofuels, pharmaceuticals and other processed products [4,5,6]. Forest plantations are part of the global biomass which is associated with the bulk of the Earth’s biological diversity [3]. They act as biological filters and are associated with the water and nutrient cycles; they are also active absorbers of carbon dioxide and carbon, thus supporting microbial, animal and plant biodiversity [5,6,7].

During their life cycle, trees maintain close relationships with representatives of various microbial communities, all of which together represent the plant microbiota [8]. Each tree is a unique long-living organism that can be considered as an individual ecosystem densely populated by various microorganisms and exposed to abiotic, biotic and anthropogenic factors [9,10,11,12].

With the development of trade and industrial relations between countries, pathogenic microorganisms, including bacteria, have become more widespread, posing a serious threat to important tree species in horticulture, urban landscaping and silviculture [13,14,15]. In addition, forest trees are reservoirs of bacteria that affect both forest plants and economically valuable woody and herbaceous plants.

Most research today is devoted to studying bacterial diseases affecting fruit trees, while studies of forest plants are conducted to a much lesser extent [16]. The etiology of the large number of currently known forest tree diseases has not yet been clarified. Among the most studied are bacterial blight diseases caused by *Erwinia amylovora*, bacterial wilt diseases caused by a complex of *Ralstonia solanacearum* species, root and stem galls on trees caused by *Agrobacterium tumefaciens*, bacterial scald caused by *Xylella fastidiosa*, bacterial canker caused by *Pseudomonas syringae* and wetwood disease caused by bacteria from the following genera: *Pseudomonas*, *Pantoea*, *Xanthomonas*, *Klebsiella*, *Enterobacter*, *Clostridium*, *Bacillus* [16,17,18]. Bacterial diseases that damage oak stands include bacterial leaf streak caused by *Xylella fastidiosa*, acute oak decline caused by *Gibbsiella quercinecans*, *Rahnella victoriana*, and *Brenneria goodwinii* and drippy nut disease caused by *Brenneria quercina* (formerly *Erwinia quercina*) [19].

A number of studies have been devoted to the study of bacterial diseases of eucalyptus trees (*Eucalyptus* spp). It was found that the symptoms of bacterial spotting, interveinal necrosis and shoot dieback in Australia, Brazil and Uruguay were caused by bacteria of the genus *Xanthomonas* [20,21], although in Brazil similar symptoms were also caused by representatives of the genera *Pseudomonas*, *Rhizobiaceae* and *Erwinia* [22,23]. In southern Africa, damage to the vascular tissue of young shoots and leaves of eucalyptus which led to the destruction of new plantings was caused by *Ralstonia solanacearum* [24].

Another disease described for forest trees is bacterial canker. On wild hazelnuts (*Corylus avellana* L.), it was found to be caused by *Pseudomonas avellanae*, leading to wilting and dieback of branches [25]. Bacterial dropsy accompanied by the release of methane-containing juice from macerating tissues and complete drying of the tree is widespread on coniferous and deciduous trees throughout Russia and neighboring countries [26,27,28]. This disease is usually associated with bacteria of the genera *Erwinia*, *Enterobacter*, *Pseudomonas* and *Dickeya* [29].

Despite the fact that bacterial diseases of forest trees play a significant role in forest pathology, their distribution and etiology in Russia remain virtually unstudied.

## 2. Results

### 2.1. Bacterial Characterization

**Figure 1:**
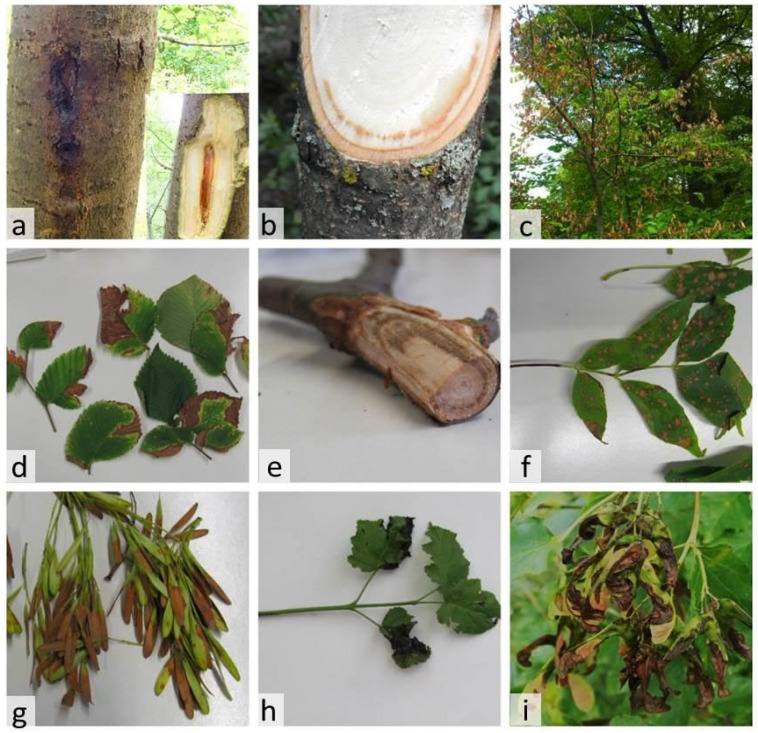
Symptoms exhibited on bacterial-infected forest trees. (**a**) Wet spot on the trunk with wood browning on *P. tremula*; (**b**) peripheral wood vessel browning on *U. laevis*; (**c**) wilt of *U. laevis* crown with symptoms of necrotic spots on leaves and browning of areas of peripheral and central wood; (**d**) necrotic spots on leaves of *U. laevis*; (**e**) peripheral wood vessel browning on *U. minor*; (**f**) necrotic spots on leaves of *F. pennsylvanica*; (**g**) necrotic spots on winged fruits of *F. pennsylvanica*; (**h**) necrotic spots on leaves of *A. tataricum*; (**i**) necrotic spots on winged fruits of *A. tataricum*.

As a result of phytosanitary monitoring, we collected samples from 28 trees with symptoms of leaf spotting and wood darkening, which were preliminarily associated with phytopathogenic bacteria (Figure 1, Supplementary Table S1). From these samples, we purified 48 isolates of Gram-negative bacteria, 29 of which were similar in phenotype to *Pseudomonas* bacteria and 19 to *Pantoea* bacteria (Supplementary Figure S1).

All *Pseudomonas* isolates had an oxidative type of glucose metabolism, were catalase-positive, negative in the indol production test, grew at +4 °C and did not grow at 37 °C. Of the tested carbon sources, the *Pseudomonas* isolates utilized galactose, mannitol, inositol and aspartic acid. Sorbitol was utilized by all isolates except 236v2, whereas sucrose was not utilized by isolates 159v4 and 230d4 only. The ability to utilize arabinose, lactate, rhamnose, trehalose and tartrate depended on the isolate (Supplementary Table S2).

Based on the LOPAT tests, the *Pseudomonas*-like isolates were divided into two groups. Group Ia (which comprised *Pseudomonas syringae*) included isolates that formed light beige colonies on the KingB and YDC media and fluoresced on KingB (however, some isolates lost this ability over time). These colonies were levan-positive, oxidase-negative, arginine dehydrolase-negative, caused HSR on tobacco and showed no pectolytic activity on potatoes.

Group Ib (which comprised various *Pseudomonas* species, including *P. delphini*) included two isolates, 159v4 and 230d4, that did not fluoresce on KingB and formed light beige colonies after the first inoculation, but became yellow after several passes. On the YDC medium, the colonies were yellow. The colonies formed a clear zone around them as a result of the dissolution of CaCO_3_ due to the intense production of acid during glucose utilization. These colonies were levan-negative, oxidase-negative, arginine dehydrolase-negative, did not cause soft rot on potatoes but caused HSR on tobacco.

All *Pantoea* isolates had yellow mucoid colonies on the YDC medium. A clear zone formed around the colonies of isolate 39m1 due to the dissolution of CaCO_3_. Colonies did not fluoresce on the KingB medium. All isolates had a fermentative type of glucose metabolism, were catalase-positive, negative in the indol production test and grew at +4 °C. All isolates, except 152a1 and 197.2, grew at +37 °C. The *Pantoea* colonies were levan-negative, oxidase-negative, arginine dehydrolase-negative, caused HSR on tobacco and showed no pectolytic activity on potatoes. Of the tested carbon sources, the *Pantoea* isolates utilized sucrose, galactose, trehalose, mannitol, inositol and aspartic acid. Rhamnose was utilized by all isolates except 252v1. The ability to utilize arabinose, lactate, sorbitol and tartrate depended on the isolate.

### 2.2. Identification Using 16S rRNA Gene

Based on the 16S rRNA gene sequencing data, 29 isolates were identified as *Pseudomonas* and 19 isolates as *Pantoea*. For all isolates, the shared identity with the nucleotide sequences from the GenBank database was more than 99%.

**Figure 2:**
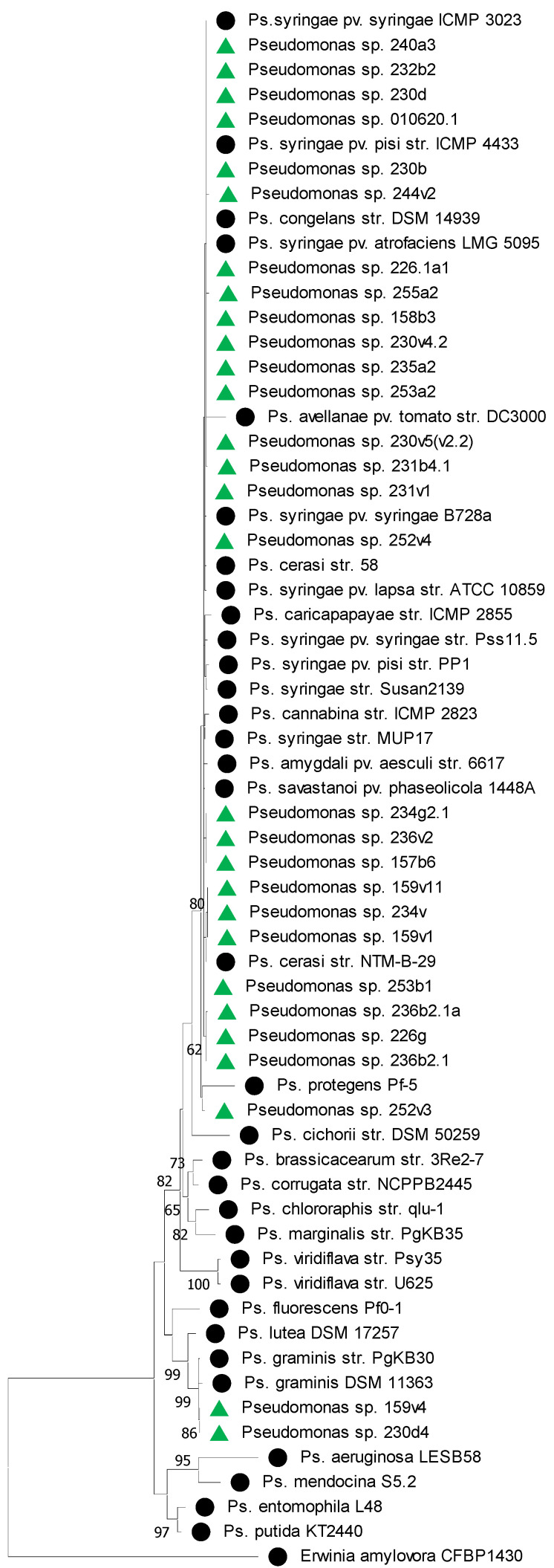
Phylogenetic relationships of *Pseudomonas* sp. based on the partial 16S rRNA gene sequence. The tree was constructed in the MEGA 11 program using the neighbor-joining method. The bootstrap analysis of 1000 runs. Green triangles indicate isolates from this study. Black circles indicate reference strains from the NCBI database. The strain *Erwinia amylovora* CFBP1430 was used as an outgroup.

On the phylogenetic tree, the *Pseudomonas* isolates divided into two clusters (Figure 2). One of them included 24 isolates which, based on their biochemical properties, belonged to group Ia and were classified as *P. syringae*. This cluster also included isolates of other *Pseudomonas* species, the nucleotide sequences of which were taken from the GenBank database. The second cluster included two isolates which, based on their biochemical properties, were assigned to group Ib. The closest isolates from the GenBank database with a bootstrap support of 100 were *P. graminis*.

**Figure 3:**
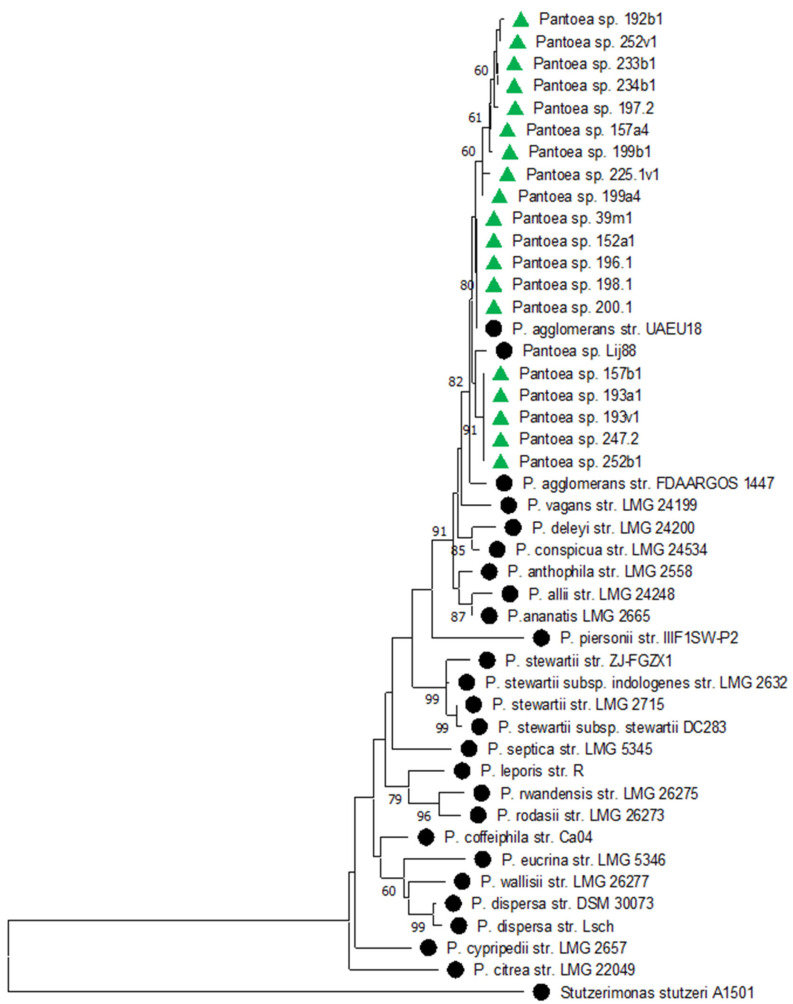
Phylogenetic relationships of *Pantoea* sp. based on the partial 16S rRNA gene sequence. The tree was constructed in the MEGA 11 program using the neighbor-joining method. The bootstrap analysis of 1000 runs. Green triangles indicate isolates from this study. Black circles indicate reference strains from the NCBI database. The strain *Stutzerimonas stutzeri* A1501 was used as an outgroup.

On the phylogenetic tree, all *Pantoea* isolates clustered closely to each other. The closest isolates from the GenBank database were *P. agglomerans* and *P. vagans* (Figure 3).

### 2.3. Multilocus Typing

A concatenated dataset for the analysis of *Pseudomonas* isolates was constructed from partial nucleotide sequences of five housekeeping genes, *gapA*, *gltA*, *gyrB*, *rpoD* and *rpoB,* with lengths of 533, 686, 674, 752 and 1154 bp, respectively. The length of the concatenated sequence comprised 3799 bp.

**Figure 4:**
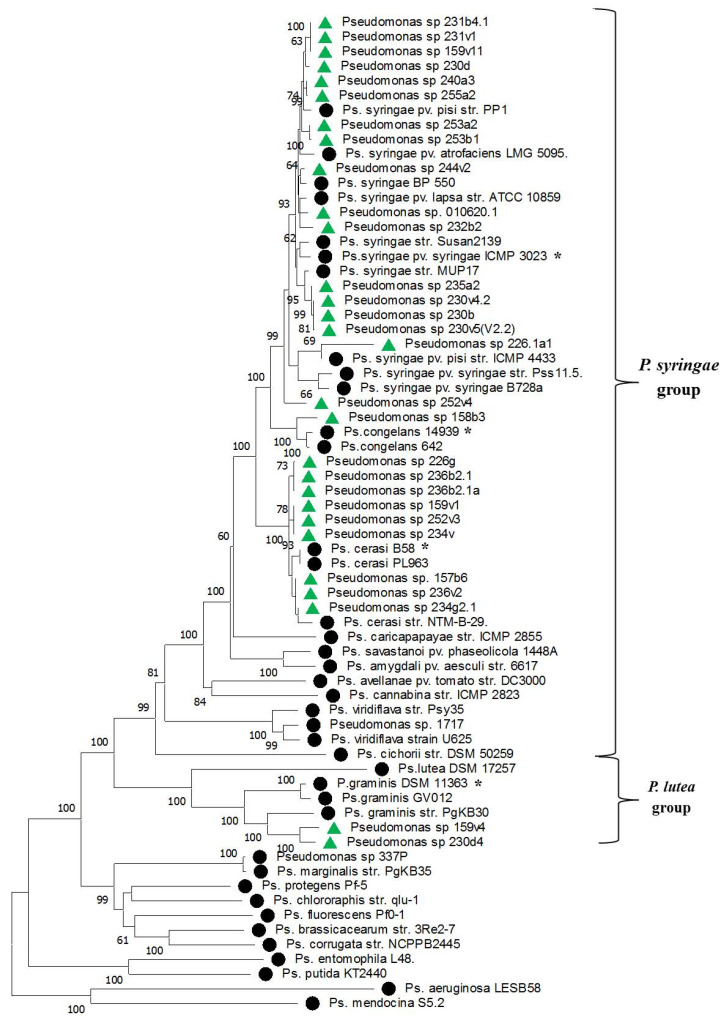
Phylogenetic relationships of *Pseudomonas* sp. based on typing data of concatenated sequences of 5 genes: *gapA*, *gltA*, *gyrB*, *rpoD* and *rpoB*. The tree was constructed in the MEGA 11 program using the neighbor-joining method. Bootstrap analysis of 1000 runs. Green triangles indicate isolates from this study. Black circles indicate reference strains from the NCBI database. Stars indicate typical strains of species.

In the concatenated phylogeny of *Pseudomonas*, the strains identified by us divided into two clusters. One of them included isolates 159v4 and 230d4 which clustered near the *P. graminis* reference isolate and formed the *P. lutea* phylogenetic group. The other cluster included a phylogenetic group of *P. syringae* synonymous species together with reference isolates of *P. syringae*, *P. caricapapayae*, *P. cerasi* and *P. congelans* (Figure 4).

Based on the combination of biochemical characteristics, a phylogenetic analysis based on the 16S rRNA gene and MLST, isolates 159v4 and 230d4 were identified as *P. graminis*, isolates 226g, 236b2.1, 236b2.1a, 157b6, 236v2, 234g2.1, 234v, 159v1, 252v3 as *P. cerasi*, isolate 158b3 as *P. congelans*, and all other *Pseudomonas* isolates as *P. syringae* (Table 1).

Based on the Tajima statistics, the most polymorphic gene of *Pseudomonas* was shown to be *gyrB*. The П value for the isolates identified by us was 0.057, while for the sequence selection supplemented with reference isolates it was 0.099 (Table 2). At the same time, the Tajima’s D values were positive (0.32 and 0.499, respectively). High values of nucleotide diversity were also determined for the *gapA* (П = 0.044 for the isolates identified by us, П = 0.096 for the selection supplemented with reference isolates) and *rpoD* (П = 0.033 and П = 0.093, respectively) genes. However, the Tajima’s D value for both genes was negative, which indicates low divergence within the gene (Table 2).

It is interesting that the Tajima’s D value for the *rpoB* gene for the isolates identified by us was negative (−1.215), while it was positive (0.119) in the selection with reference isolates. The nucleotide diversity index was 0.022 and 0.063, respectively. A phylogenetic analysis based on the *rpoB* gene did not allow us to classify the isolates identified by us as a pathovariant of *P. syringae* (Supplementary Figure S2).

As a result of the study conducted on the selection of Russian isolates, the nucleotide diversity of Pseudomonas comprised 0.005 (0.5%), and that of *Pantoea* was 0.003 (0.3%). The corresponding index in the selection supplemented with the reference isolates comprised 0.014 and 0.019, respectively (Table 2).

Removing the nucleotide sequences of the *gyrB*, *gapA*, *rpoD* and *rpoB* genes from the phylogenetic analysis one by one did not affect the clustering of the isolates on the dendrogram, but led to a decrease in the bootstrap support (Supplementary Figures S3–S6).

To analyze the *Pantoea* isolates, we created a concatenated dataset based on partial nucleotide sequences of six housekeeping genes, *gyrB*, *rpoB*, *fusA*, *leuS*, *pyrG* and *rplB,* with lengths of 574, 992, 742, 741, 404 and 449 bp, respectively. The length of the concatenated sequence comprised 3902 bp.

**Figure 5:**
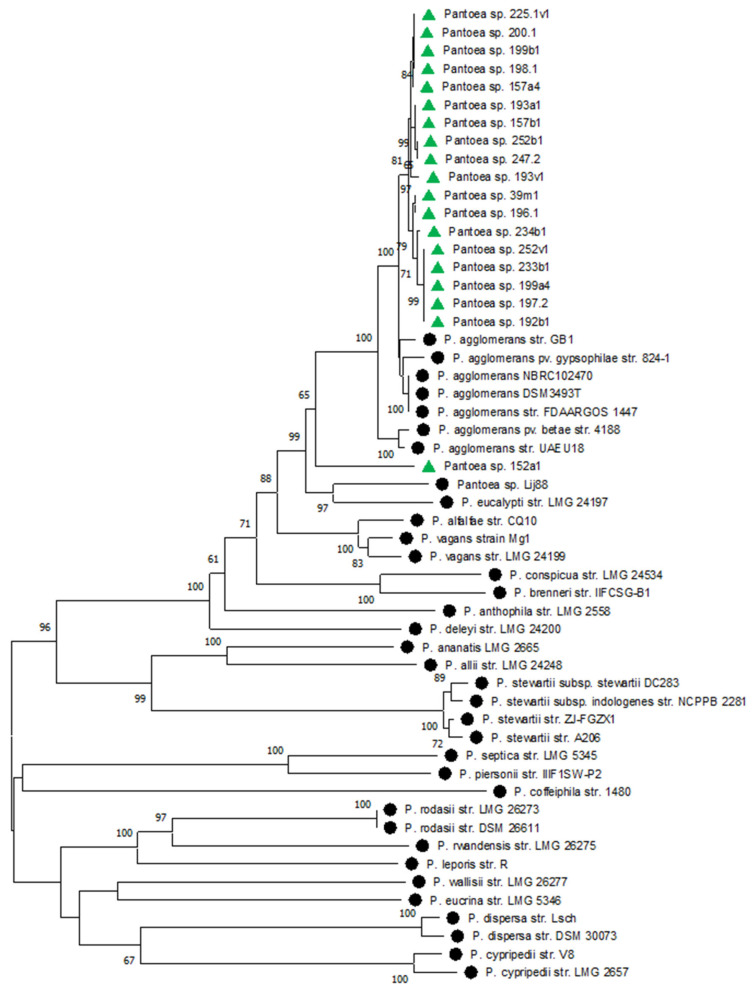
Phylogenetic relationships of *Pantoea* based on typing data of concatenated sequences of 6 genes: *gyrB*, *rpoB*, *fusA*, *leuS*, *pyrG*, *rplB*. The tree was constructed in the MEGA 11 program using the neighbor-joining method. Bootstrap analysis of 1000 runs. Green triangles indicate isolates from this study. Black circles indicate reference strains from the NCBI database.

The main part of the concatenated *Pantoea* sequences on the dendrogram were grouped into a separate cluster consisting only of the isolates identified in this study (Figure 5). The closest reference isolates were *P. agglomerans* with a bootstrap support of 100. Isolate 152a1 which clustered with *P. agglomerans* based on the 16S rRNA gene was allocated to a separate branch on the concatenated phylogenetic tree between the clusters with *P. agglomerans* and *P. eucalypti*. Based on biochemical characteristics, as well as the results of the phylogenetic analysis for the 16S rRNA gene and MLST, the isolates of *Pantoea* purified from *Acer* sp., *Ulmus* sp. and *Fraxinus* sp. were identified as *P. agglomerans*. For more a accurate identification of the *Pantoea* sp. isolate 152a1 purified from *P. tremula*, additional studies are required (Table 1).

An analysis of the Tajima statistics showed that the nucleotide diversity values of six genes in the isolates we identified were lower than those in the analysis for the selection supplemented with reference isolates, while Tajima’s D values were negative (Table 2). At the same time, the Tajima’s D values for *leuS* (−2.14) *and pyrG (*−2.16) were lower than those for other genes. The highest nucleotide diversity values were determined for the *leuS* (П = 0.122) and *gyrB* (П = 0.109) genes in the expanded selection, while Tajima’s D values were positive (1.03 for *leuS* and 1.13 for *gyrB*). The lowest П values in both selections were shown for the *rplB* gene (П = 0.002 for the isolates identified by us and П = 0.034 for the selection supplemented with reference isolates) with negative Tajima’s D values (d = −1.525 and d = −0.23, respectively). Removing the nucleotide sequences of the *leuS* and *gyrB* genes from the phylogenetic analysis one by one did not affect the clustering of isolates (Supplementary Figures S7 and S8).

### 2.4. Pathogenicity Tests

**Figure 6:**
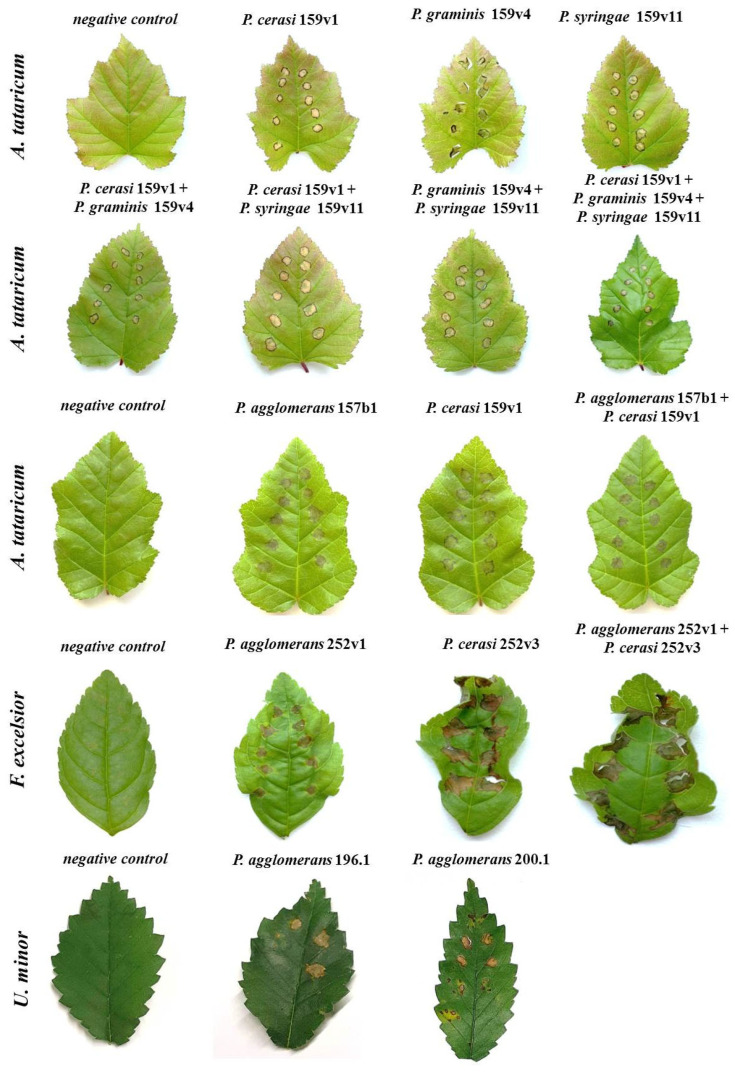
Pathogenicity test for the isolates of *Pseudomonas* sp. and *Pantoea* sp. on seedlings of different forest trees.

As a result of the injection of a bacterial suspension into the leaves of *A. tataricum*, *U. minor* and *F. pennsylvanica*, necroses began to develop on all tested plants within 24 h after inoculation. Necroses formed both after inoculation with a monoculture of *P. cerasi* 159v1, *P. graminis* 159v4, *P. syringae* 159v11, *P. cerasi* 252v3, *P. agglomerans* 157b1, *P. agglomerans* 196.1, *P. agglomerans* 200.1 and *P. agglomerans* 252v1 and after inoculation with a mixture of two or three *Pseudomonas* isolates, as well as a mixture of *Pantoae* and *Pseudomonas* (Table 3, Figure 6). Inoculation with water did not result in the appearance of any symptoms. All reisolated isolates were identical in phenotype and nucleotide sequence of the 16S rRNA gene to the isolates used for inoculation.

It is interesting that, after inoculation with a mixture of bacteria, the ratio of reisolated colonies of *Pantoae* and *Pseudomonas* from two plant species, *A. tataricum* and *F. pennsylvanica*, was unequal: per 300 colonies of *Pantoae*, there were about 2–5 colonies of *Pseudomonas*, which suggests that the growth of bacteria of the genus *Pseudomonas* was suppressed by representatives of the genus *Pantoea* (Supplementary Figure S9).

## 3. Discussion

In this study, we describe for the first time new etiological agents associated with forest tree diseases: leaf spotting and wood darkening. Leaf spotting on *A. tataricum* and *F. pennsylvanica* was caused by *P. syringae*, *P. congelans*, *P. cerasia* and *P. agglomerans* both as a monoinfection and in various combinations. It is worth noting that while genetic diversity was observed among *Pseudomonas* even within one examined location and they were represented by isolates of the same or different species, almost all *Pantoea* were represented by closely related isolates of *P. agglomerans*.

Most of the analyzed *Ulmus* sp. samples and one *P. tremula* sample had symptoms of wood darkening. These plants were found to be infected only with *P. agglomerans*, the pathogenicity of which was confirmed on the leaves of *Ulmus* sp. According to our data, this bacterial species was isolated from forest trees with the symptoms of wood darkening for the first time. The etiological agents of leaf spotting and wood darkening were found to be the same in both examined locations, the distance between which exceeded 250 km.

Coinfection of forest trees with *Pseudomonas* and *Pantoea* indicates a complex interaction mechanism between two bacterial populations. When confirming Koch’s postulates and re-isolating bacteria after infection with two or more isolates, we noted a significantly larger number of *Pantoea* colonies than *Pseudomonas*. Previously, the mechanism of interaction between these two bacteria, based on mutual stimulation and growth suppression, had been described for the olive knot disease, the successful development of which depends on the amount of inoculum of each of the bacteria [30,31]. It has also been found that coinfection of several pathogens can cause more harm to the host plant than monoinfection [31]. The detailed role of each of the pathogenic isolates that we identified in the development of the infection and the specifics of their interaction are yet to be established.

In this study, we identified *Pseudomonas* and *Pantoea* isolates that were similar in terms of their morphological, biochemical and physiological characteristics. The lack of strict standards for the classification of bacteria species complicated establishing taxonomic and evolutionary affiliation, which the authors of other studies also encountered [32,33]. For instance, a phenotypic characterization of *Pseudomonas* isolates based on LOPAT diagnostic tests allowed us to identify two groups of pathogenic bacteria classified as *P. syringae* (group Ia) and *P. graminis* (group Ib). Subsequently, MLST made it possible to identify two phylogenetic groups of species: the *P. syringae* group which included the synonymous species *P. syringae*, *P. congelans* and *P. cerasi*, and the *P. lutea* group which included *P. graminis*. It should be noted that MLST showed a better resolution for the species identification of *Pseudomonas* and *Pantoea* compared to taxonomic affiliation based on the 16S rRNA gene. The low values of 16S rRNA gene polymorphism obtained as a result of Tajima’s statistics analysis confirm the efficiency of using this gene for the reconstruction of intergeneric phylogenetic relationships, which is consistent with the results of previous studies [34,35,36,37]. Thus, an integrated approach based on describing phenotypic, biochemical and phylogenetic characteristics allows for the most accurate classification of the detected bacterial isolates. The authors of other studies on bacterial identification came to the same conclusion [38,39,40,41].

As a result of the analysis of the Tajima’s statistic indices for *Pseudomonas*, the D value for the *gapA*, *gltA*, *gyrB*, *rpoD* and *rpoB* genes was found to range from −1.47 to 0.499. This indicates a lack of rare alleles of these genes in the analyzed populations and, as a consequence, the presence of neutral evolution. Therefore, changes in the nucleotide sequences of the five genes do not affect the adaptability of *Pseudomonas* isolates to environmental conditions. The Tajima’s D index for the *gyrB*, *rpoB*, *fusA* and *rplB* genes of all analyzed *Pantoea* isolates ranged from −1.7 to 1.13, which indicates the presence of neutral evolution of these genes. For the *leuS* and *pyrG* genes of the *Pantoea* isolates that we identified, the Tajima’s D index was −2.14 and −2.16, respectively, which suggests the presence of positive selection for these isolates. However, the same index for the extended selection of isolates was 1.03 and 0.646, respectively, indicating the presence of neutral evolution. This suggests that Tajima’s D values largely depend on the analyzed selection; therefore, when analyzing the type of gene evolution, the selection should include representative isolates. Thus, the results of phylogenetic analysis and statistical analysis of Tajima’s D values indicate the need for careful selection of genes for the reconstruction of the phylogenetic relationships within each study, since genes that are informative for one species may not be informative enough for other species. Similar conclusions have been made in other studies [42,43].

This study describes *Pseudomonas* and *Pantoea* isolates, which are pathogens both for economically valuable herbaceous plants and for woody plants: fruit and forest trees. Therefore, forest trees can be a reservoir of dangerous diseases, including bacterial ones. Further study of the species composition, distribution and specifics of the pathogenesis of the identified bacteria should become the basis for developing methods for their control and preventing the development of epiphytotics.

## 4. Materials and Methods

### 4.1. Locations and Sample Collection

The study was conducted at two sites located in the forest–steppe zone of the European part of Russia: in the Tellerman experimental forestry (Voronezh Oblast) and the Galichya Gora Nature Reserve (Lipetsk Oblast).

The Tellerman experimental forestry is located on the bank of the Khopyor River [44] (51°21′ N, 42°00′ E). The age of the Tellerman forest is estimated to be approximately 7.6 thousand years [45]. Locations with 200–270-year-old oak groves of natural origin are protected areas. Since 1944, researchers have been conducting there biogeocenotic, silvicultural and ecological studies, as well as studies on the biodiversity of the ecosystems of broad-leaved forests, within the southern forest–steppe [44,46,47,48,49,50,51]. The tree layer is formed by common ash (*Fraxinus pennsylvanica* L.), English oak (*Quercus robur* L.), small-leaved linden (*Tilia cordata* Mill.) and Norway maple (*Acer platanoides* L.).

The Galichya Gora Nature Reserve is located in a refugium area with a large number of relict plants [52,53]. Conservation activity has been carried out there since 1925. Sampling was carried out by us at the Galichya Gora and Morozova Gora sites which are unique territories with a long history of scientific research. The Galichya Gora site is located on the right bank of the Don River (52°36′ N, 38°55′ E), and the Morozova Gora site is located on the left bank of the Don River (52°36′ N, 38°56′ E). The tree layer is formed by *Quercus robur*, *Betula sp.* and *A.tataricum* [54,55]. Samples with symptoms of necrotic leaf and fruit spotting were collected from trees of four species: Tatar maple (*A. tataricum*)—18 samples; European white elm (*U. laevis*)—6 samples; field elm (*U. minor*)—1 sample; Pennsylvania ash (*F. pennsylvanica*)—2 samples; and common aspen (*P. tremula*)—1 sample in the summer of 2020. Samples with symptoms of wood darkening were collected in August 2018 and 2020 from European white elm *U. laevis* in the Tellerman forest. The collected samples were placed in individual bags, stored at a temperature close to +4 °C and delivered to the laboratory for analysis.

### 4.2. Isolation of Bacteria

Fragments of leaf, petiole and fruit tissues from the edges of necrotic lesions measuring approximately 0.5 cm^2^ were surface sterilized in 0.5% KMnO_4_ solution for 1 min, washed twice in sterile distilled water and incubated for 15 min in 300 μL of water. Then, 50 μL of the suspension were plated on the LB nutrient medium and incubated at 28 °C [56]. After 24–48 h, single colonies were reseeded by the depletion method. The resulting pure bacterial cultures were stored at −70 °C.

### 4.3. Phenotypic and Biochemical Characterization of Bacterial Strains

For phenotypic characterization and biochemical testing, bacteria were grown on the KingB nutrient medium at 28 °C for 12–24 h. All tests were performed in duplicate using control strains with known properties.

The biochemical, physiological and cultural properties of bacterial isolates (determination of colony type and catalase activity, Gram staining, ability to grow at +4 °C and +37 °C, ability to produce indole from tryptophan, type of glucose metabolism) were characterized as described previously [57,58]. The ability of colonies to fluoresce in UV light was determined on the KingB medium, and the yellow pigment formation was assessed on the YDC medium supplemented with calcium carbonate [58].

For species identification of *Pseudomonas* isolates, the LOPAT test system was used [59], including levan production tests, determination of oxidase activity, pectolytic activity on potatoes, arginine dihydrolase production and hypersensitivity reactions on tobacco. Further characterization of the isolates was carried out based on their ability to utilize carbon from eleven sources of sugars and polyhydric alcohols: arabinose, lactose, sucrose, rhamnose, glucose, sorbitol, trehalose, tartrate, mannitol, inositol and aspartic acid [58].

### 4.4. Molecular Characterization of Bacterial Strains Based on 16S rDNA

Bacteria were grown on the KingB nutrient medium for 24 h. Single colonies were transferred to microcentrifuge tubes, supplemented with sterile water and incubated at 95 °C for 10 min [30]. The resulting bacterial DNA samples were stored at −70 °C. Then, 1 μL of the lysate was used as a template for PCR amplification of the 16S rRNA gene with the primers listed in Supplementary Table S3. The reaction mixture contained 1x Taq Buffer with (NH4)_2_SO_4_, 0.2 mM each dNTP, 1 μM each primer, 2.5 mM MgCl_2_, 0.375 U Taq polymerase (Thermo Fisher Scientific, Waltham, MA, USA). PCR was performed on a T100 Thermal Cycler (BioRad, Hercules, CA, USA) according to the following program: initial denaturation at 95 °C for 2 min, then 35 cycles of 95 °C for 40 s, 56 °C for 40 s and 72 °C for 90 s. The final elongation step was performed at 72 °C for 5 min. The obtained PCR products were separated in 1% agarose gel; fragments of about 1500 bp were cut out and purified using the Cleanup Standard Kit according to the manufacturer’s protocol (Eurogen, Moscow, Russia). The isolated fragments were sequenced by the Sanger method using the Big Dye Terminator v.3.1 chemical reagents on an ABI PRIZM 3730 automatic sequencer according to the manufacturer’s instructions. Nucleotide sequences were aligned and assembled using the MEGA 11 software [60] and used for BLASTn analysis against the GenBank and EzBioCloud databases. The sequences were deposited in GenBank, and their accession numbers are provided in Supplementary Table S4.

### 4.5. Multilocus Phylogenetic Analysis

Multilocus sequence typing (MLST) was performed on the housekeeping genes of the bacteria. For the analysis of *Pseudomonas* strains, fragments of 5 genes were amplified and sequenced: *gapA* (the gene encoding glyceraldehyde-3-phosphate dehydrogenase), *gltA* (the gene encoding citrate synthase), *gyrB* (the gene encoding DNA gyrase B), *rpoD* (the gene encoding the σ-subunit of RNA polymerase) and *rpoB* (the gene encoding the β-subunit of RNA polymerase). For MLST of Pantoea strains, fragments of 6 genes were used: *gyrB* (the gene encoding the β-subunit of DNA gyrase), *rpoB* (the gene encoding the β-subunit of RNA polymerase), *fusA* (the gene encoding elongation factor G), *leuS* (the gene encoding leucyl-tRNA synthase), *pyrG* (the gene encoding CTP synthase) and *rplB* (the gene encoding large ribosomal subunit protein uL2) [61,62]. For amplification, primers specific to the corresponding locus were used (Supplementary Table S3). PCR products were sequenced in both directions. Complete nucleotide sequences of the genes were assembled as described above and compared with the reference sequences available in the GenBank and EzBioCloud databases using BLASTn analysis. Alignment was performed for each locus using the ClustalW algorithm with default settings. All sequences were trimmed and used to create a concatenated dataset and perform the phylogenetic analysis.

In addition to the concatenated four-gene dataset, for *Pseudomonas* we added *rpoB* which has been used in the literature to classify closely related strains into pathovariants [63,64].

Phylogenetic trees were constructed using the Neighbor-Joining (NJ) method with a bootstrap support of 1000 replicates in the MEGA 11 program [60]. Sequences of type strains of *Pseudomonas* and *Pantoea* species from LPSN (Supplementary Tables S5 and S6) were used as references. To root the trees, the nucleotide sequences of *Stutzerimonas stutzeri* A1501 (NC_009434.1) and *Erwinia amylovora* CFBP1430 (NC_013961.1) were used.

The polymorphism of the genes used for MLST was assessed using the Tajima’s test. For this, the nucleotide diversity (П)) and the Tajima’s d index were assessed in two sequence selections. One selection included only the isolates obtained in this work. The second selection was supplemented with reference sequences of various species of *Pseudomonas* (or *Pantoae*) from the GenBank database. To assess the effect of polymorphic genes on the reconstruction of phylogenetic relationships, we removed the most polymorphic genes one by one from the concatenated sequences, and then analyzed the clustering results. The nucleotide sequences were deposited in GenBank (Supplementary Table S4).

### 4.6. Characterization of Pseudomonas Isolates Based on rpoB

The characterization of *P. syringae* pathovariants was carried out based on the nucleotide sequence of a fragment of the *rpoB* gene encoding the β-subunit of the RNA polymerase [32,64]. PCR was performed with the primers listed in Supplementary Table S3. The assembled nucleotide sequences were used to construct a phylogenetic tree using the NJ method with a bootstrap support of 1000 replicates. All reference strains used in this study are listed in Supplementary Table S4.

### 4.7. Pathogenicity Tests

To satisfy Koch’s postulates for the isolated *Pseudomonas* and *Pantoea* strains, we performed inoculation of host plants and re-isolation of bacteria from leaves with symptoms of infection. For testing, we used young, well-developed plants of the same species from which the bacteria were isolated.

Pure cultures of representative strains were grown for 48 h at 28 °C, suspended in sterile water and used to prepare inoculums with cell concentrations of 10^8^ cells/mL. When inoculation was carried out with a mixture of strains, their ratio in the suspension was equal. As a control, sterile water was used instead of bacterial suspension. Infections were carried out in a greenhouse at 25 °C. The manifestation of symptoms was monitored daily for 14 days after inoculation.

Inoculation of seedling leaves was performed in a greenhouse in two technical replicates through injection of the bacterial suspension using a medical syringe into the underside of the leaf in 10 places. Repeated isolation of the bacteria was performed from leaf fragments with symptoms of infection. Pure bacterial cultures obtained as described above that were phenotypically similar to the original inoculum strain were analyzed using PCR with primers for the 16S rRNA gene (Supplementary Table S3).

## Figures and Tables

**Table 1:** Pathogenic bacteria *Pseudomonas and Pantoea* identified on woody species.

Symptoms	Plant Species	Plant Number	Bacterial Species and Isolate	Sampling Location	Date of Sampling
Leaf spot	*Acer tataricum*	10620.1	*P. syringae* 10620.1	Tellerman	22/06/2020
		157	*P. agglomerans* 157a4, 157b1	Galichya Gora	08/07/2020
			*P. cerasi* 157b6		
		158	*P. congelans* 158b3		
		159	*P. cerasi* 159v1		
			*P. graminis* 159v4		
			*P. syringae* 159v11		
		192	*P. agglomerans* 192b1	Tellerman	20/07/2020–24/07/2020
		193	*P. agglomerans* 193a1, 193v1		
		225	*P. agglomerans* 225.1v1	Galichya Gora	21/08/2020–22/08/2020
		226	*P. syringae* 226.1a1		
			*P. cerasi* 226g		
		230	*P. syringae* 230b, 230v4.2, 230v5 (v2.2), 230d		
			*P. graminis* 230d4		
		231	*P. syringae* 231b4.1, 231v1		
		232	*P. syringae* 232b2		
		233	*P. agglomerans* 233b1		
		234	*P. agglomerans* 234b1		
			*P. cerasi* 234v, 234g2.1		
		235	*P. syringae* 235a2		
		236	*P. cerasi* 236b2.1, 236b2.1a, 236v2		
		240	*P. syringae* 240a3	Tellerman	24/08/2020–30/08/2020
		244	*P. syringae* 244v2		
		255	*P. syringae* 255a2		
	*Ulmus minor*	247	*P. agglomerans* 247.2		
	*Fraxinus pennsylvanica*	252	*P. agglomerans* 252b1, 252v1		
			*P. cerasi* 252v3		
			*P. syringae* 252v4		
		253	*P. syringae* 253a2		
	*Ulmus laevis*	196	*P. agglomerans* 196.1		20/07/2020–24/07/2020
Darkening of wood	*Ulmus laevis*	39	*P. agglomerans* 39m1		05/08/2018
	*Populus tremula*	152	*Pantoea* sp. 152a1	Galichya Gora	08/07/2020
	*Ulmus laevis*	197	*P. agglomerans* 197.2	Tellerman	20/07/2020–24/07/2020
		198	*P. agglomerans* 198.1		
		199	*P. agglomerans* 199a4, 199b1		
		200	*P. agglomerans* 200.1		

**Table 2:** Tajima’s statistics for the isolates of *Pseudomonas* and *Pantoea*.

*Pseudomonas*			
Genes	Set of Isolates	П (%)	Tajima’s D
*gapA*	this research	0.044	−0.71
	this research + representative	0.096	−0.024
*gltA*	this research	0.026	−0.6
	this research + representative	0.056	−0.54
*gyrB*	this research	0.057	0.32
	this research + representative	0.099	0.499
*rpoD*	this research	0.03	−1.47
	this research + representative	0.093	−0.645
*rpoB*	this research	0.022	−1.215
	this research + representative	0.063	0.119
16S	this research	0.005	−1.559
	this research + representative	0.014	−1.29
***Pantoea***			
*gyrB*	this research	0.011	−1.48
	this research + representative	0.109	1.13
*rpoB*	this research	0.003	−1.7
	this research + representative	0.072	0.789
*fusA*	this research	0.003	−1.62
	this research + representative	0.064	0.596
*leuS*	this research	0.013	−2.14
	this research + representative	0.122	1.03
*pyrG*	this research	0.002	−2.16
	this research + representative	0.067	0.646
*rplB*	this research	0.002	−1.525
	this research + representative	0.034	−0.23
16S	this research	0.003	1.48
	this research + representative	0.019	0.093

**Table 3:** *Pantoae* and *Pseudomonas* isolates selected for pathogenicity tests on *A. tataricum*, *U. minor* and *F. pennsylvanica*.

Plant Species	Testing Isolates	Test Result
*Acer tataricum*	*P. cerasi* 159v1	+
	*P. graminis* 159v4	+
	*P. syringae* 159v11	+
	*P. cerasi* 159v1 *+ P. graminis* 159v4	+
	*P. cerasi* 159v1 *+ P. syringae* 159v11	+
	*P. graminis* 159v4 *+ P. syringae* 159v11	+
	*P. cerasi* 159v1 *+ P. graminis* 159v4 *+ P. syringae* 159v11	+
	*H_2_O*	-
*Acer tataricum*	*P. agglomerans* 157b1	+
	*P. cerasi* 159v1	+
	*P. agglomerans* 157b1 *+ P. cerasi* 159v1	+
	*H_2_O*	-
*Fraxinus pennsylvanica*	*P. agglomerans* 252v1	+
	*P. cerasi* 252v3	+
	*P. agglomerans* 252v1 *+ P. cerasi* 252v3	+
	*H_2_O*	-
*Ulmus minor*	*P. agglomerans* 196.1	+
	*P. agglomerans* 200.1	+
	*H_2_O*	-

## Data Availability

All data are presented in the manuscript and Supplementary Materials. Further details are available on request from the corresponding authors.

## Supplementary Materials

The following supporting information can be downloaded at: https://www.mdpi.com/article/10.3390/plants14040563/s1, Figure S1: Colony morphology of *Pantoea* and *Pseudomonas* isolates on LB medium; Figure S2: Phylogenetic relationships of *Pseudomonas* based on partial sequence of the *rpoB*; Figure S3: Phylogenetic relationships of *Pseudomonas* based on typing data of concatenated sequences of 4 genes: *gapA*, *gltA*, *rpoD*, *rpoB*; Figure S4: Phylogenetic relationships of *Pseudomonas* based on typing data of concatenated sequences of 4 genes: *gltA*, *gyrB*, *rpoD*, *rpoB*; Figure S5: Phylogenetic relationships of *Pseudomonas* based on typing data of concatenated sequences of 4 genes: *gapA*, *gltA*, *gyrB*, *rpoB*; Figure S6: Phylogenetic relationships of *Pseudomonas* based on typing data of concatenated sequences of 4 genes: *gapA*, *gltA*, *gyrB*, *rpoD*; Figure S7: Phylogenetic relationships of *Pantoea* based on typing data of concatenated sequences of 5 genes: *rpoB*, *fusA*, *leuS*, *pyrG*, *rplB*; Figure S8: Phylogenetic relationships of *Pantoea* based on typing data of concatenated sequences of 5 genes: *gyrB*, *rpoB*, *fusA*, *pyrG*, *rplB*; Figure S9: Reisolation of bacteria from *A. tataricum*; Table S1: Symptoms and bacterial isolates; Table S2: Physiological and biochemical characterization of bacterial isolates; Table S3: Primers and PCR conditions; Table S4: Amplified sequences of bacterial genes uploaded into GenBank with their identifier; Table S5: Reference strains for MLST of *Pseudomonas* sp; Table S6: Reference strains for MLST of *Pantoea* sp.
