# Supplementary material for: Bacteria Pseudomonas sp. and Pantoea sp. Are the New Etiological Agents of Diseases on Forest Trees
Source: Plants (Basel). 2025 Feb 12;14(4):563. doi: 10.3390/plants14040563 (PMC11859248; doi:10.3390/plants14040563)
Supplement: Supplementary file 1 [file plants-14-00563-s001.zip › Supplementary Figures.pdf]

Supplementary materials

Porotikova et al., 2024. Bacteria *Pseudomonas* sp. And *Pantoea* sp. Are the New Etiological Agents of Diseases on Forest Trees

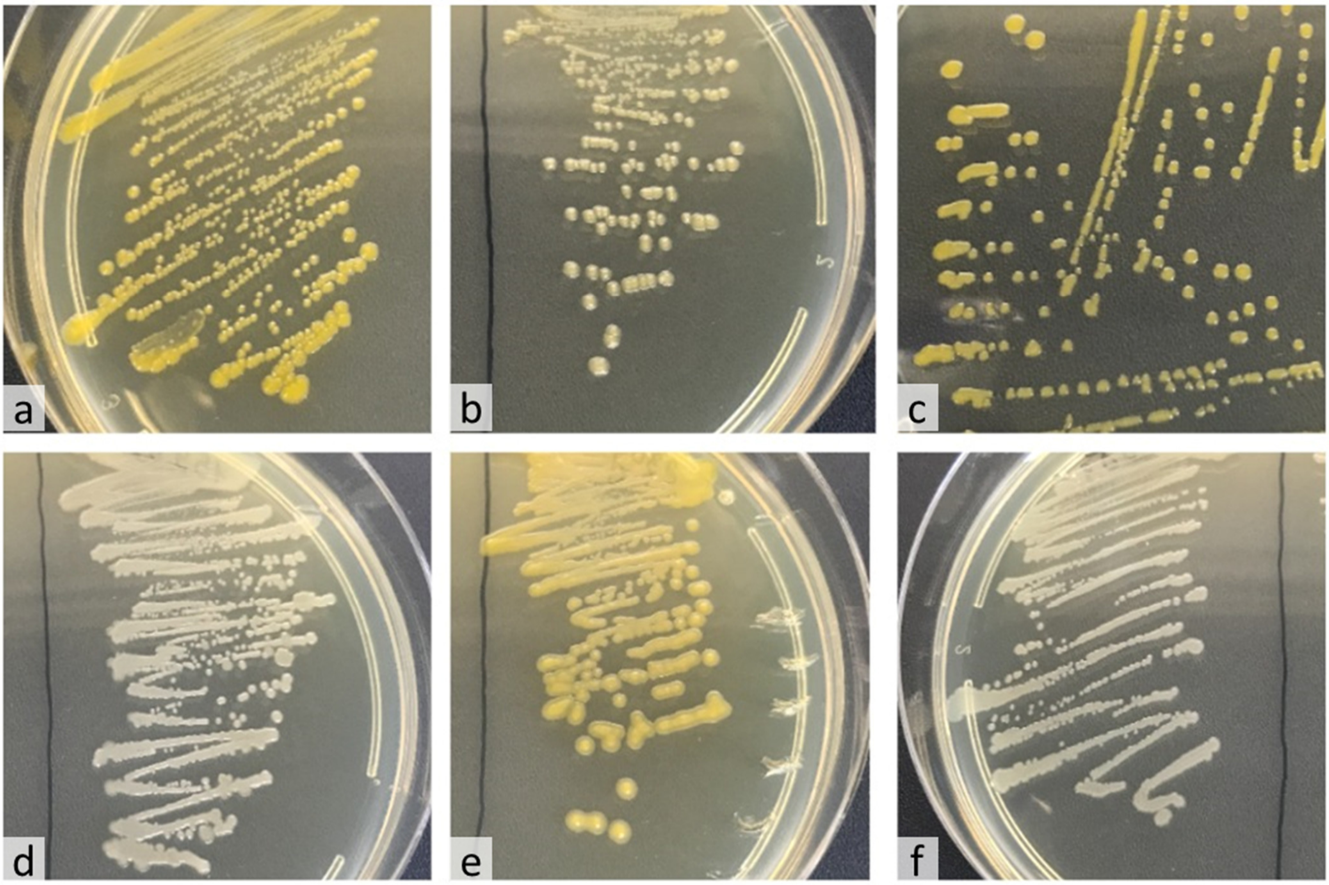

Figure S1. Colony morphology of *Pantoea* and *Pseudomonas* isolates on LB medium. **(a)** *Pantoea agglomerans* 157a4; **(b)** *P. agglomerans* 39m1; **(c)** *P. agglomerans* 152a1; **(d)** *Pseudomonas cerasi* 159v1; **(e)** *Pseudomonas graminis* 230d4; **(f)** *Pseudomonas congelans* 158b3

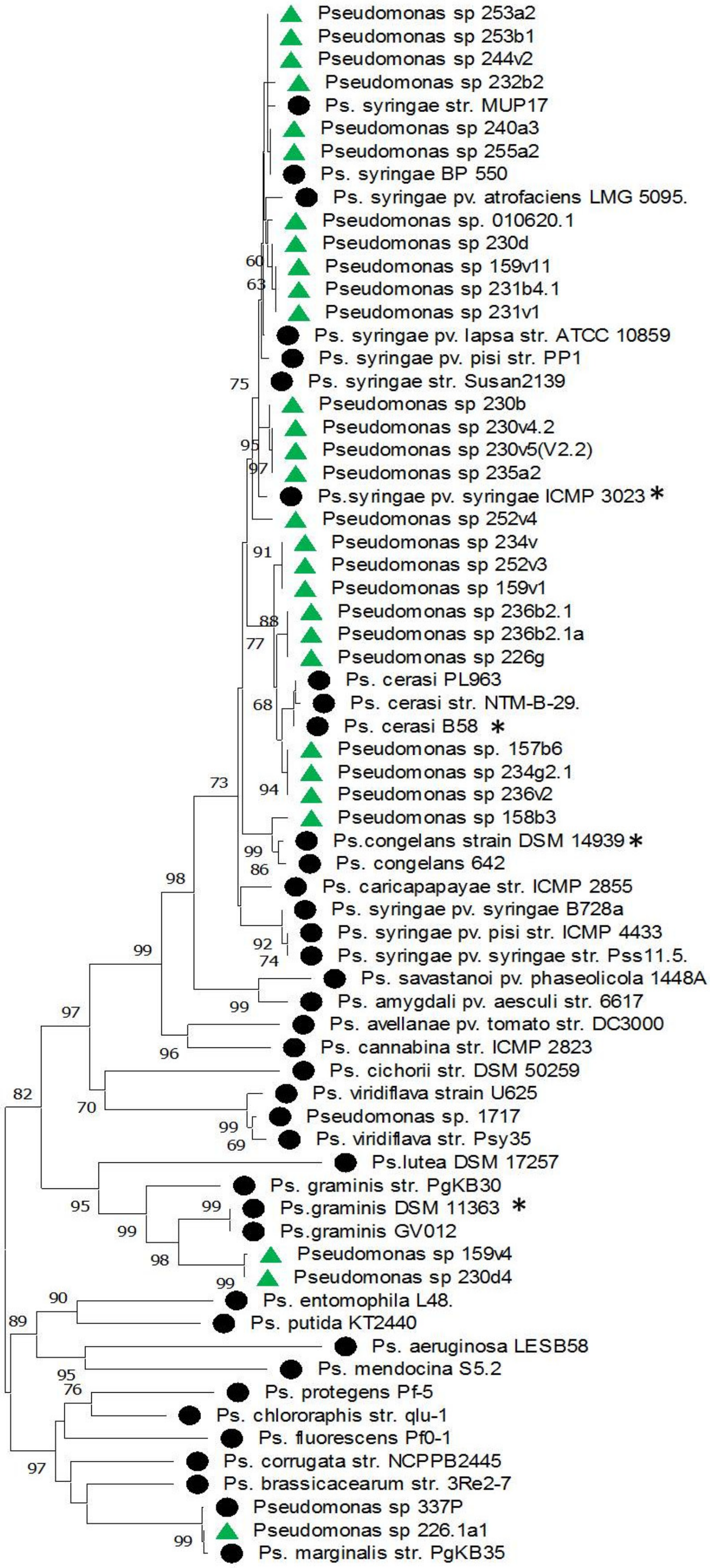

Figure S2. Phylogenetic relationships of *Pseudomonas* based on partial sequence of the *rpoB*. The tree was constructed in the MEGA 11 program using the Neighbor-Joining (NJ) method. Bootstrap analysis of 1,000 runs. Green triangles indicate isolates from this study, black circles indicate reference strains from the NCBI database, stars indicate typical strains of species

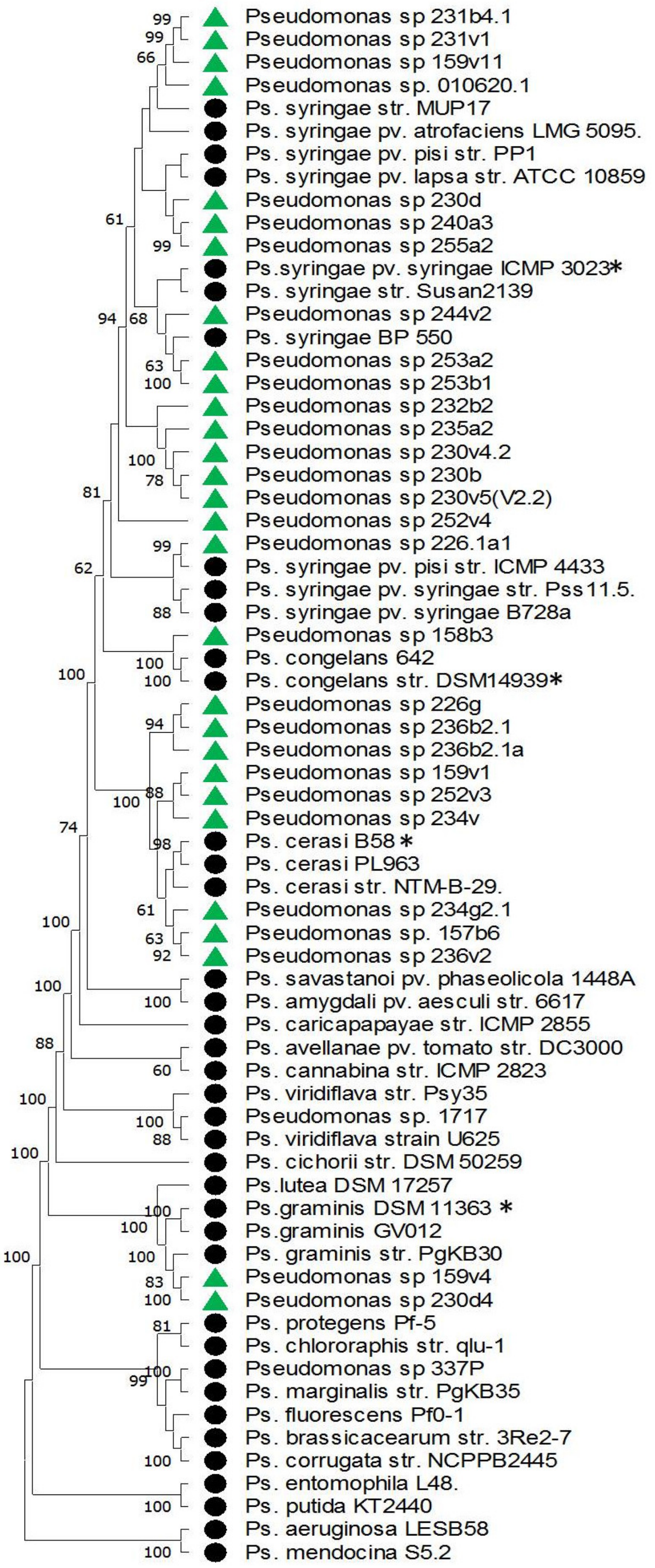

Figure S3. Phylogenetic relationships of *Pseudomonas* based on typing data of concatenated sequences of 4 genes: *gapA, gltA, rpoD, rpoB*. The tree was constructed in the MEGA 11 program using the Neighbor-Joining (NJ) method. Bootstrap analysis of 1,000 runs. Green triangles indicate isolates from this study,

black circles indicate reference strains from the NCBI database, stars indicate typical strains of species

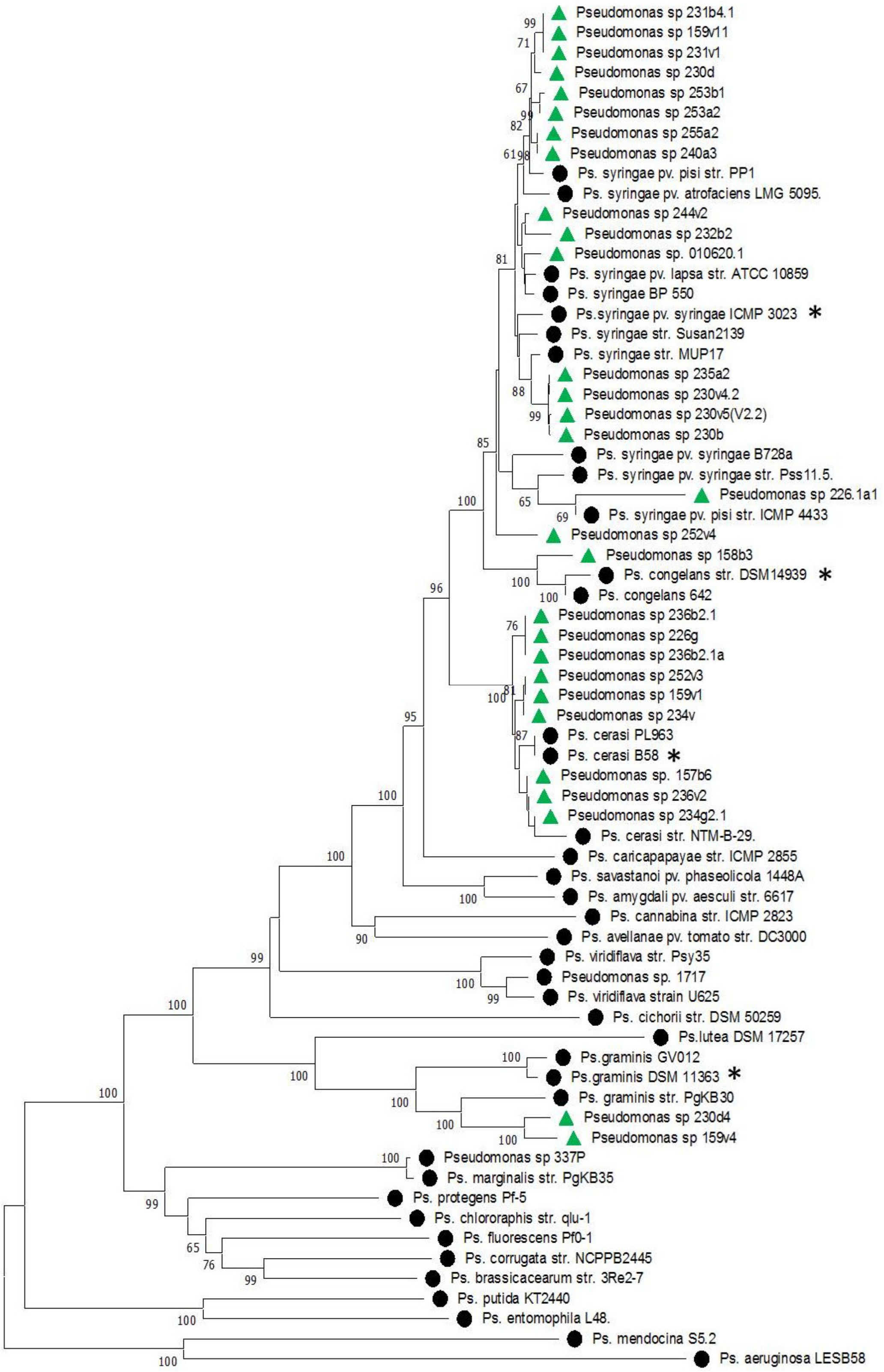

Figure S4. Phylogenetic relationships of *Pseudomonas* based on typing data of concatenated sequences of 4 genes: *gltA, gyrB, rpoD, rpoB*. The tree was constructed in the MEGA 11 program using the Neighbor-Joining (NJ) method. Bootstrap analysis of 1,000 runs. Green triangles indicate isolates from this study, black circles indicate reference strains from the NCBI database, stars indicate typical strains of species

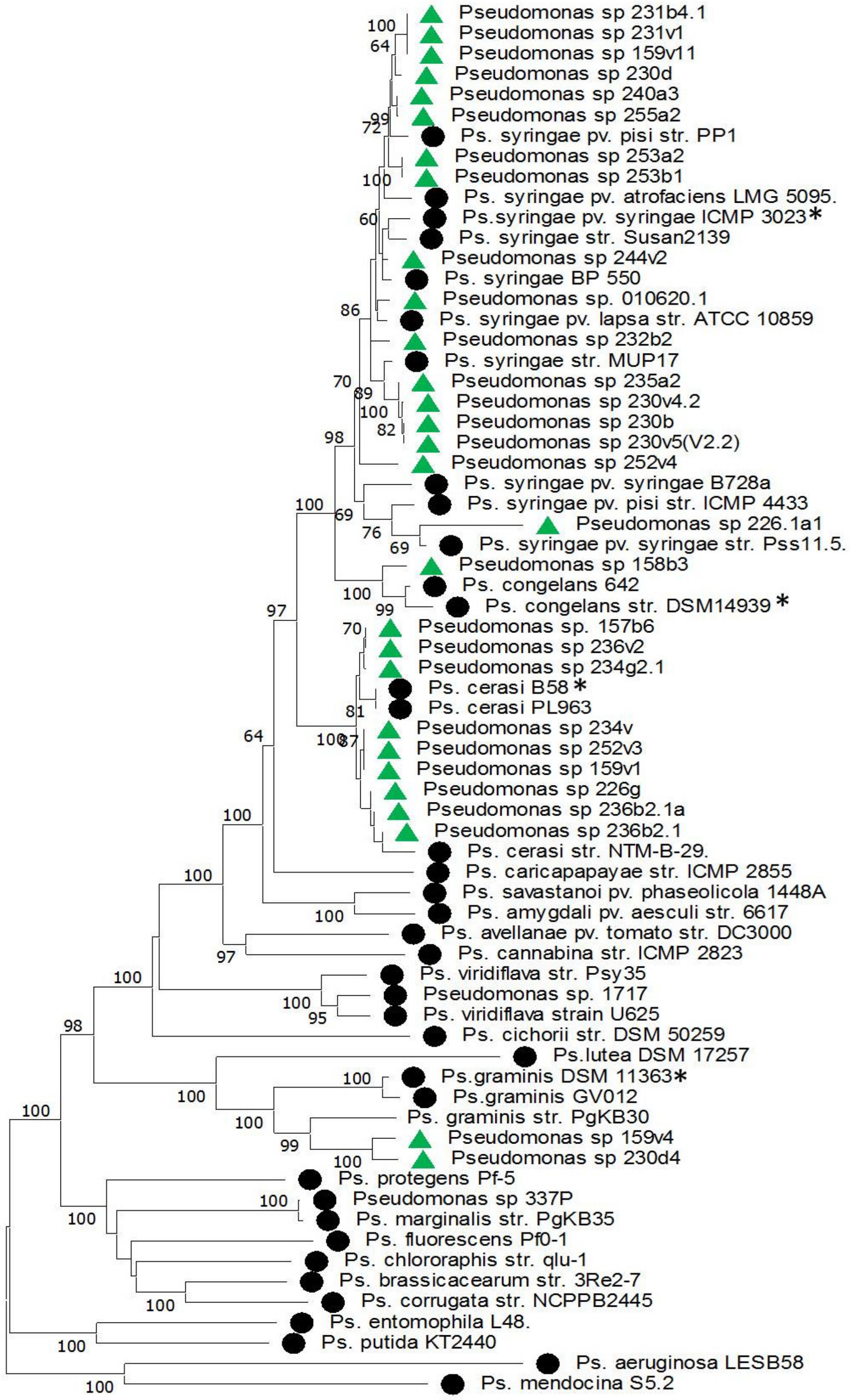

Figure S5. Phylogenetic relationships of *Pseudomonas* based on typing data of concatenated sequences of 4 genes: *gapA, gltA, gyrB, rpoB*. The tree was constructed in the MEGA 11 program using the Neighbor-Joining (NJ) method. Bootstrap analysis of 1,000 runs. Green triangles indicate isolates from this study,

black circles indicate reference strains from the NCBI database, stars indicate typical strains of species

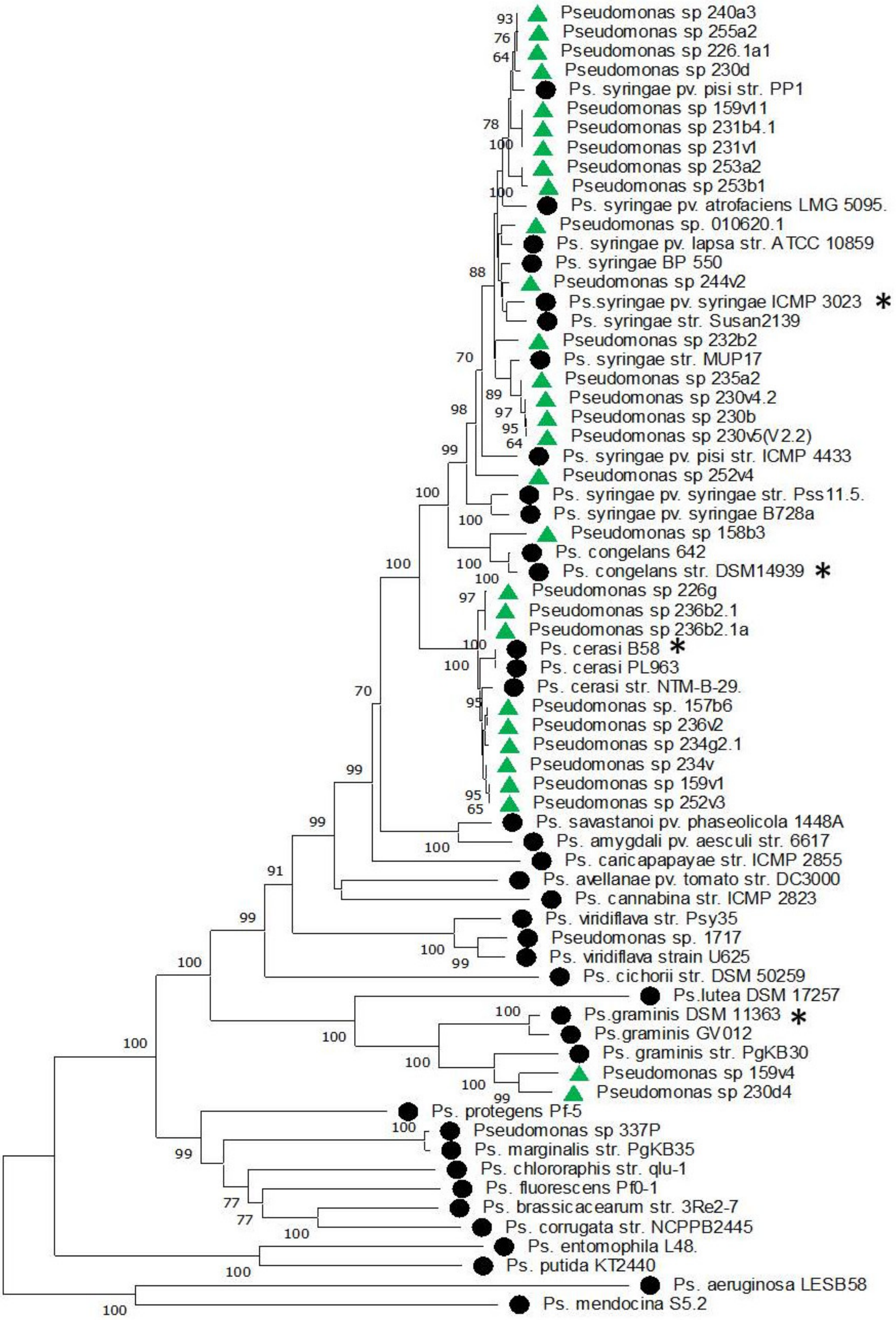

Figure S6. Phylogenetic relationships of *Pseudomonas* based on typing data of concatenated sequences of 4 genes: *gapA, gltA, gyrB, rpoD*. The tree was constructed in the MEGA 11 program using the Neighbor-Joining (NJ) method. Bootstrap analysis of 1,000 runs. Green triangles indicate isolates from this study,

black circles indicate reference strains from the NCBI database, stars indicate typical strains of species

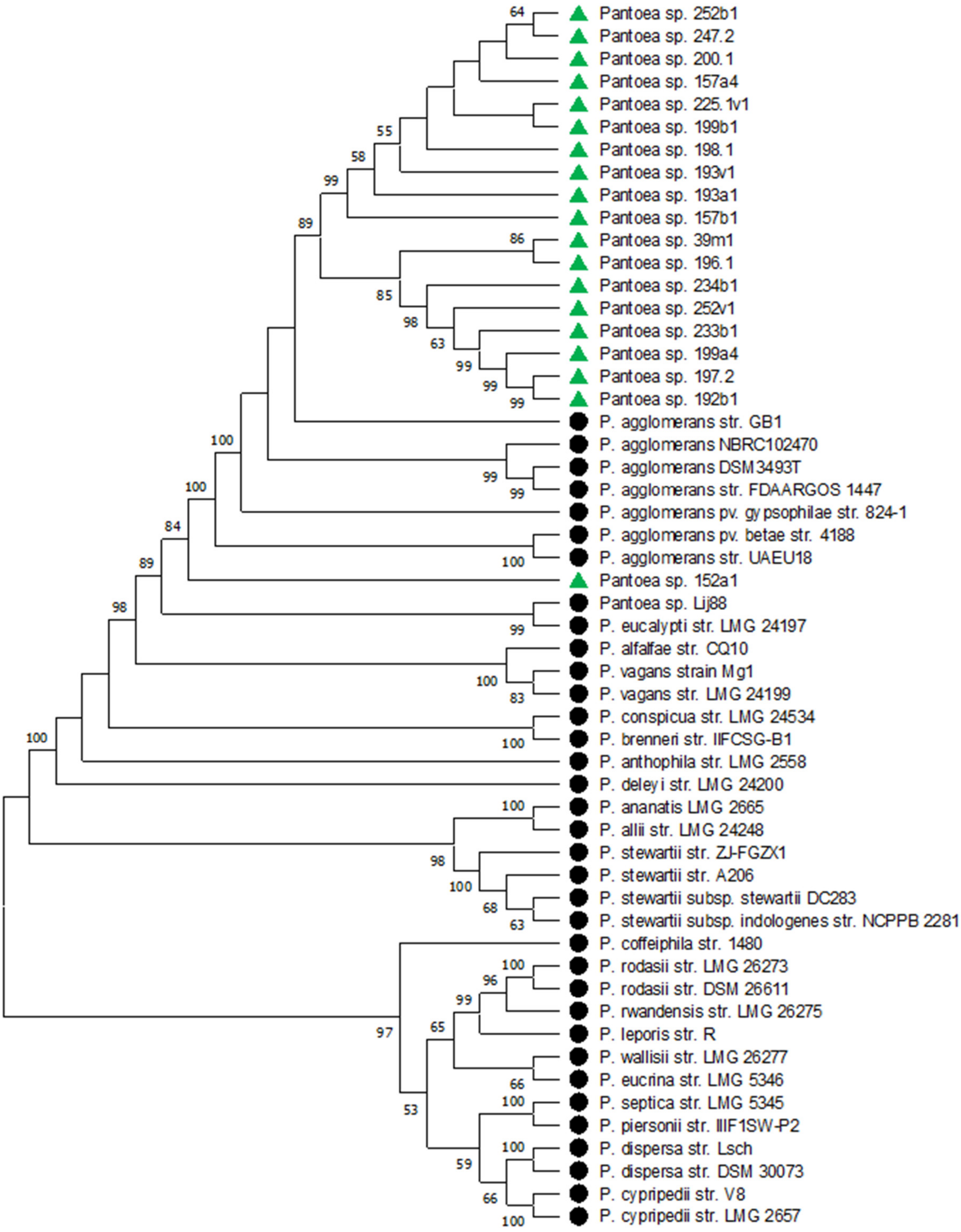

Figure S7. Phylogenetic relationships of *Pantoea* based on typing data of concatenated sequences of 5 genes: *rpoB, fusA, leuS, pyrG, rplB*. The tree was constructed in the MEGA 11 program using the Neighbor-Joining (NJ) method. Bootstrap analysis of 1,000 runs. Green triangles indicate isolates from this study, black circles indicate reference strains from the NCBI database

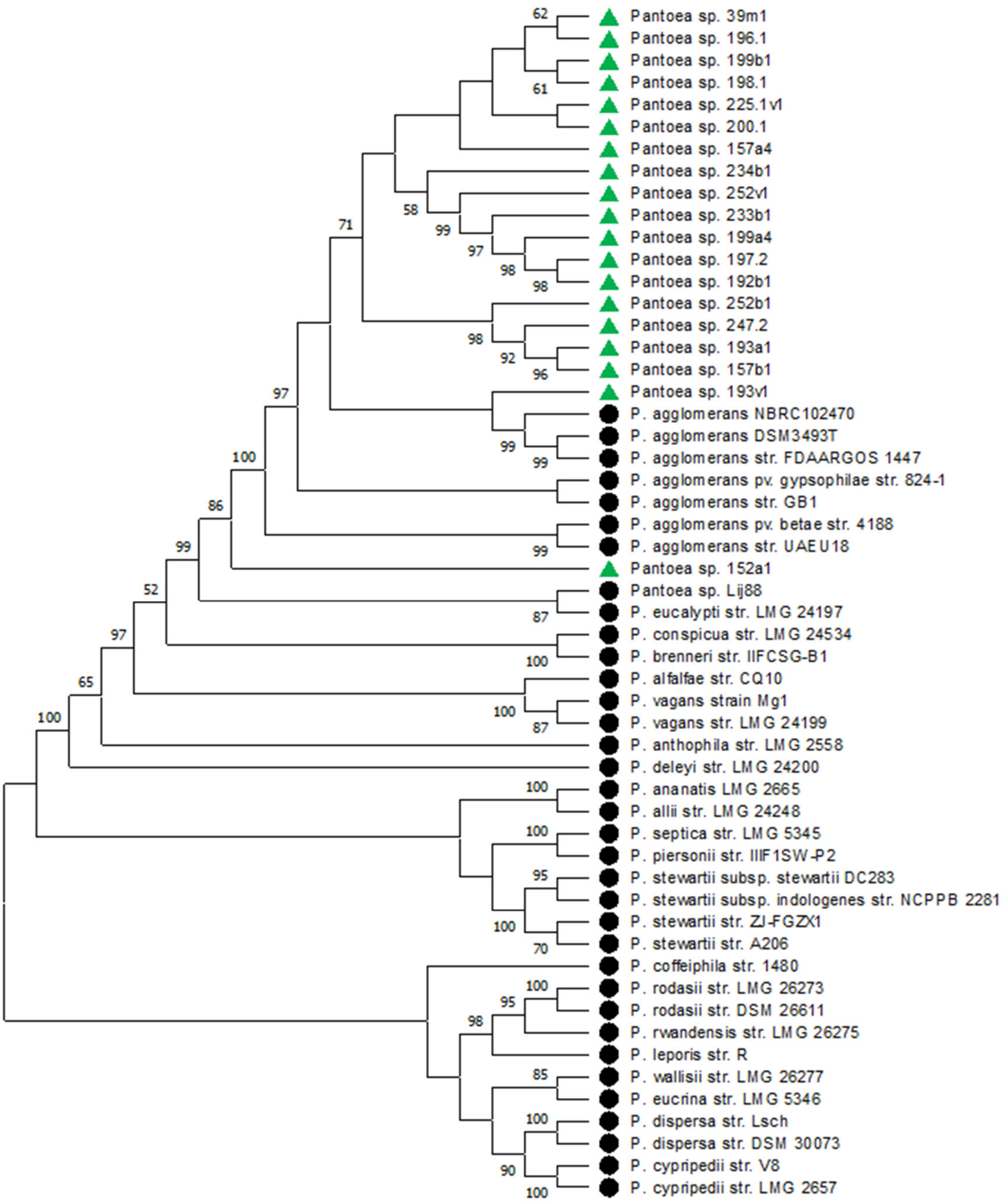

Figure S8. Phylogenetic relationships of *Pantoea* based on typing data of concatenated sequences of 5 genes: *gyrB, rpoB, fusA, pyrG, rplB*. The tree was constructed in the MEGA 11 program using the Neighbor-Joining (NJ) method. Bootstrap analysis of 1,000 runs. Green triangles indicate isolates from this study, black circles indicate reference strains from the NCBI database

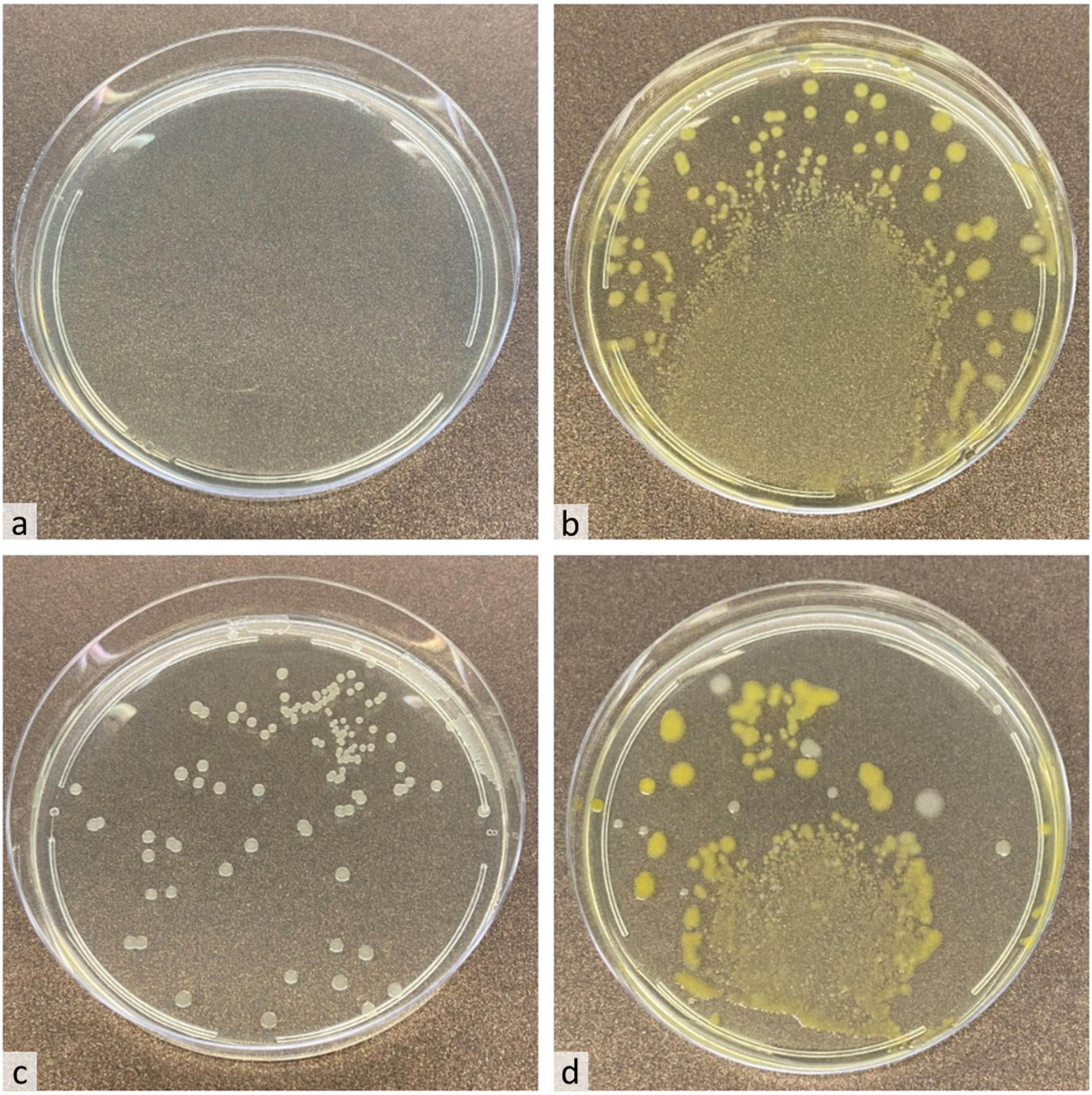

Figure S9. Reisolation of bacteria from *A. tataricum*. a - negative control (inoculation with water), b - *P. agglomerans* 157b1, c - *P. cerasi* 159v1, d - *P. agglomerans* 157b1+*P. cerasi* 159v1
